# Fabrication of microfluidic device for Aflatoxin M1 detection in milk samples with specific aptamers

**DOI:** 10.1038/s41598-020-60926-2

**Published:** 2020-03-13

**Authors:** Aruna Kasoju, Deepshikha Shahdeo, Azmat Ali Khan, Narlawar Sagar Shrikrishna, Subhasis Mahari, Amer M. Alanazi, Mashooq Ahmad Bhat, Jyotsnendu Giri, Sonu Gandhi

**Affiliations:** 1 DBT- National Institute of Animal Biotechnology, Hyderabad, 500032 India; 2 Department of Biotechnology, JNTUA College of Engineering, Andhra Pradesh, 516390 India; 3 Department of Pharmaceutical Chemistry, College of Pharmacy, Kind Saud University, Riyadh, 11451 Kingdom of Saudi Arabia; 4 Department of Biomedical Engineering, Indian Institute of Technology (IIT), Hyderabad, 502285 India

**Keywords:** Biological techniques, Nanoscience and technology

## Abstract

This study describes the colorimetric detection of aflatoxin M1 (Afl M1) in milk samples using a microfluidic paper-based analytical device (µPAD). Fabrication of µPADs was done using a simple and quick approach. Each μPAD contained a detection zone and a sample zone interconnected by microchannels. The colorimetric assay was developed using unmodified AuNPs as a probe and 21-mer aptamer as a recognition molecule. The free aptamers were adsorbed onto the surface of AuNPs in absence of Afl M1, even at high salt concentrations. The salt induced aggregation of specific aptamers occurred in presence of Afl M1. Under optimum conditions, the analytical linear range was found to be 1 µM to 1 pM with limit of detection 3 pM and 10 nM in standard buffer and spiked milk samples respectively. The proposed aptamer based colorimetric assay was repeatable, quick, selective, and can be used for on-site detection of other toxins in milk and meat samples.

## Introduction

Mycotoxins are secondary metabolites produced by filamentous fungi belonging to the genera Aspergillus and Penicillium^1^. Mycotoxins are also found in animal derived foods such as milk due to intake of contaminated feed^2,3^.

Considering agricultural and economical aspects and possible implications on public health, the most relevant mycotoxins are aflatoxins, ochratoxins, fuminosins, T-2 toxin, and zearalenone (ZEA)^4^. Aflatoxins pose a huge economic burden as they cause around 25% or more loss of the world’s food crops every year^5^. Afl M1 (4-hydroxy aflatoxin B1) and M2 (4-dihydroxy aflatoxin B1) have well proven carcinogenic and mutagenic potentiality and pose severe health consequences on milk consumers, including the risk of cancer and stunted growth in children below the age of 5 years^6^. When B1 is ingested by a cow, it is secreted as hydroxylated metabolite aflatoxin M1 (Afl M1) in the urine and milk of the cow^7^. Consumption of food containing aflatoxin concentrations of one milligram/kilogram or higher has been suspected to cause aflatoxicosis^8^, the prognosis of which consists of acute liver failure, jaundice, lethargy, and nausea, eventually leading to death within 1 to 2 weeks, based on past reports^9^. Thus there is a need to develop rapid low cost technology based highly specific methods for detection of aflatoxins to improve surveillance and control in rural areas.

A plethora of analytical techniques are available for aflatoxin M1 detection, ranging from chromatography and HPLC-MS used for regulatory control in official laboratories to rapid test kits for grain silos and farmers, especially for surveys when outbreaks occur^10,11^. Conventional techniques for the detection of aflatoxins include gas chromatography (GC), fluorescence or UV based detection, thin layer chromatography (TLC) and enzyme linked immunosorbent assay (ELISA)^6–12^. These techniques are routinely used and yield reliable results, however they are expensive, time consuming, require large scale instrumentation and large amounts of hazardous chemical reagents. Advancement in the development of biosensors as an analytical tool for detection of pesticides, narcotic drugs, and infectious diseases promotes the importance of quick and sensitive methods of detection^13–18^. On-field and rapid detection of mycotoxins is becoming an important challenge, where biosensors may be the solution. In order to form the regulations in food industry, to ensure food safety, potential on-site novel aflatoxin detection systems including dipstick, microarray chips, hyperspectral imaging, electronic noses, molecular imprinted polymers (MIPS) and aptamer based biosensors using nanoparticles have been developed^19^. These technologies have relevance in remote areas, and resource limited developing countries like India due to its stability, ease of production, and use^20^. To meet this requirement, aptamer (as a biorecognition element) based detection has recently increased in demand, due to its higher specificity and lesser cross reactivity as compared to antibodies, as well as ease of production^12,21^. Microfluidic devices are gaining importance as cheap, mass producible, and ecofriendly which can be used as an alternative technology for on-site detection of Afl M1^22–25^.

In the present study, we have reported the detection of Afl M1 using aptamer/AuNPs (Afl M1 apt/AuNPs) complex on a paper microfluidic device. The characterization of apt/AuNPs nanocomplex (+/− Afl M1) was done by UV-Vis spectrophotometer, DLS (Dynamic Light Scattering) for hydrodynamic diameter and zeta potential measurements and TEM (Transmission Electron Microscope) for morphological and size measurements. The Afl M1 apt/AuNPs complex was formed via simple physisorption of specific aptamers onto the surface of AuNPs^26^. Afl M1 was spiked in water and milk, followed by direct application on the µPAD and change in colour was observed immediately after addition. The sensitivity was recorded by spectroscopic method (based on the absorption of AuNPs) and with naked eye observation (based on change in the color and intensity). The concentration range of Afl M1 was found to be 1 µM to 1 pM, with a detection limit (LOD) of 3 pM and 10 nM in spiked water and milk samples respectively. The developed method can be a useful tool for on-site monitoring of aflatoxin in rural areas for economically backward farmers.

## Materials and Methods

Gold (III) chloride and Aflatoxin M1 (Afl M1) were purchased from Sigma-Aldrich. Sodium citrate and sodium chloride were procured from Sisco Research Laboratories Pvt. Ltd. (SRL), India. The 21-mer aptamer sequence of Afl M1 (ACTGCTAGAGATTTTCCACAT (5′ to 3′)), The Ochratoxin aptamer sequence used was 36-mer 5-GATCGGGTGTGGGTGGCGTAAAGGGAGCATCGGACA-3 and the ssDNA oligonucleotides were synthesized by GCC biotech, India. Stock solutions (100 μM) of aptamers were dissolved in ultrapure water then stored at −20 °C. Whatman filter paper was purchased from GE healthcare, India. Trysulfonium hexaflurophosphate and Propylene glycol monomethyl ether acetate (PGMEA) were procured from Sigma-Aldrich. All reagents were of analytical grade and used as received.

### Apparatus

Absorption spectra were taken on Systonic model S-925, Single beam UV-Vis Spectrophotometer and Perkin Elmer Lambda 25 Spectrometer. Hydrodynamic diameter and zeta potential were taken using Anton-Paar Litesizer 500. Circular Dichroism (CD) spectrometer (Jasco J-1500) was used to confirm the structural orientation of the samples. TEM images were taken using JEOL-JEM 2010 operated at an accelerating voltage of 200 kV.

### Synthesis and characterization of gold nanoparticles

Gold nanoparticles were synthesized by citrate reduction method^18^. Briefly, 100 ml of 1 mM HAuCl_4_ solution was allowed to boil and 4 ml of 1% trisodium citrate was added dropwise. Change in colour to red wine was observed, indication the synthesis of citrate coated negatively charged monodispersed gold nanoparticles. AuNPs were cooled down to room temperature and characterized by UV-Vis spectroscopy, DLS and TEM.

### Optimization of salt concentration

Different concentrations (20, 40, 60, 80, 100, 180, 200 mM) of NaCl were prepared with fixed volume of AuNPs for optimization. The dilutions were incubated for 2 min and colour change was observed. The characterization of salt induced aggregation was done by UV-Vis spectroscopy, DLS, agarose gel electrophoresis, and CD. Dilutions were characterized for UV-Vis spectra, and absorbance peak ratio of A_630/520_ nm was taken to optimize the concentration of NaCl induced aggregation.

### Preparation of aptamer–modified AuNPs

Single stranded Aflatoxin M1 aptamer (21-mer) was adsorbed onto the surface of AuNPs. Different concentrations (1000, 800, 600, 400, and 200 nM) of Aflatoxin M1 aptamer were added separately with AuNPs and incubated overnight (O/N) at room temperature (RT). 400 mM of NaCl concentration was added to all Afl M1 aptamer concentrations as prepared above, to optimize the aggregation phenomenon. Solutions were incubated for 2 minutes and gradient in the colour change of AuNPs from red wine to blue was observed. The characterization was done by UV-Vis spectroscopy, DLS, agarose gel electrophoresis, and CD to obtain the optimum concentration of gold nanoparticles required for the aggregation assay using Afl M1 aptamers.

### Circular Dichroism spectrum analysis of samples

The structural changes were observed using CD spectroscopy. All three samples AuNPs, Afl M1 Apt/AuNPs and Afl M1 were prepared as described above in 1 ml of mili-Q water, and incubated at RT for 1 h. The circular dichroism of samples were scanned at a wavelength range of 220–320 nm.

### Agarose gel electrophoresis

20 ml of AuNPs were initially centrifuged at 12000 rpm for 30 min. Pellet was resuspended in 2 ml of mili-Q. 1 ml of the resuspended particles were conjugated with optimum concentration of aptamer (800 nM), 1 µM Afl M1, with and without NaCl and incubated for 2 h. All samples were loaded on 0.8% agarose gel and electrophoresis was carried out at 85 V for 90 min.

### Fabrication and preparation of paper-based microfluidic devices

Graphic design software (Autodesk AutoCAD 2019 (trial version), https://www.autodesk.in/products/autocad/overview) was used to design the microfluidic paper based analytical device (µPAD). Photoresist layer was prepared by 52% w/w negative photoresist dissolved in 5% v/v trysulfonium hexaflurophosphate salt and 43% v/v propylene glycol monomethyl ether acetate and spread uniformly on the surface of a filter paper. Paper was kept at RT for 5 min and further baked at 60 °C for 5 min. The filter paper was kept in a UV chamber (254 nm) for 30 s and allowed to cool. Further washing was done in acetone.

### Optimization of different concentration Aflatoxin M1

Aflatoxin M1 stock solution of 1 mg/ml was prepared by dissolving in 1 ml DMSO. Three- fold subsequent dilution of Afl M1 (in water) was prepared in the range of 1 µM to 1 pM. Aflatoxin M1/ Ochratoxin aptamer modified AuNPs (1000 nM) was mixed with Afl M1 (1 µM to 1 pM) and incubated for 10 min. 400 mM of NaCl was added in each vial and equilibrated for1 min and further solutions were characterized with UV-Vis spectroscopy (400–800 nm), A_630/520 nm_ ratio and DLS.

### Analytical performance for the detection of Aflatoxin M1 in Milk

Buffalo milk was purchased from a local vendor. Milk sample was defatted by centrifugation at 6,000 rpm for 15 min. Two phases of milk were separated (aqueous and oil) and the oil layer on the top was discarded. Aqueous layer of milk was taken and spiked with stock solution of aflatoxin M1 (1 μM to 1 pM). AuNPs-aptamer M1/Ochratoxin complex was mixed with prepared serial dilutions of spiked milk with Afl M1 (1 μM to 1 pM) in the presence of 400 mM NaCl. All samples were incubated for 10 min till a gradient change in colour was observed. Absorbance was taken at A_630/520 nm_ to observe the change displacement reaction between AuNPs-aptamer/Afl M1 complex.

## Results and Discussion

### Proof of concept

**Scheme 1:**
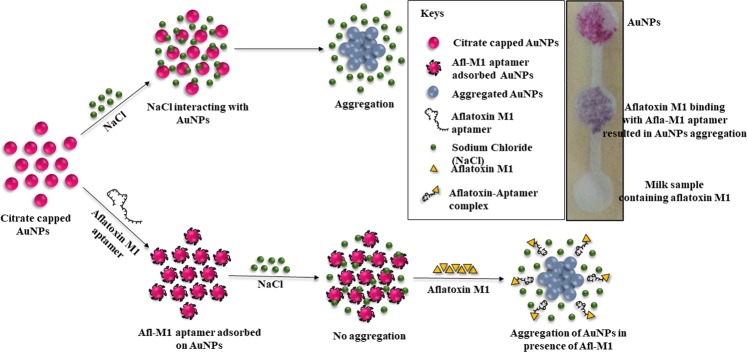
Mechanism of Aflatoxin M1 detection based on aptamer modified AuNPs. Citrate capped AuNPs showed aggregation in the presence of NaCl. AuNPs bound with specific aptamers remained dispersed in the presence of NaCl. The aggregation gets triggered after addition of Afl M1, that displaced specific aptamers and caused aggregation of AuNPs in the presence of NaCl. Inset shows the paper microfluidic device where milk samples with Afl M1 move by capillary action and blue colour developed immediately due to aggregation of specific aptamers with Afl M1, while in case of control (in absence of Afl M1 aptamer) there was no aggregation observed.

Scheme 1 shows the mechanism of how monodispersed (due to electrostatic repulsion), negatively charged AuNPs were synthesized by citrate reduction method. In the presence of an electrolyte such as NaCl, the charge repulsion between particles reduced which resulted in the aggregation of AuNPs^27,28^. Aggregation of AuNPs causes a shift in the absorbance peak to 630 nm and change in colour from wine red to blue. Aflatoxin M1 specific aptamers were adsorbed onto the surface of AuNPs by physiosorption between Au and N atom in DNA base^9^. It was observed that aptamer coated AuNPs were protected against salt aggregation. However, in the presence of Afl M1, Afl M1 aptamer dissociated from AuNPs that resulted in aggregation and colour change of the solution from wine red to blue^29^. Absorbance values of 520 nm and 630 nm represent the dispersion and aggregation phenomenon of AuNPs. Therefore, the absorbance peak ratio of A_630/520_ nm was used to determine the aggregation^26^.

### Gold nanoparticle synthesis and optimization of NaCl concentration

**Figure 1:**
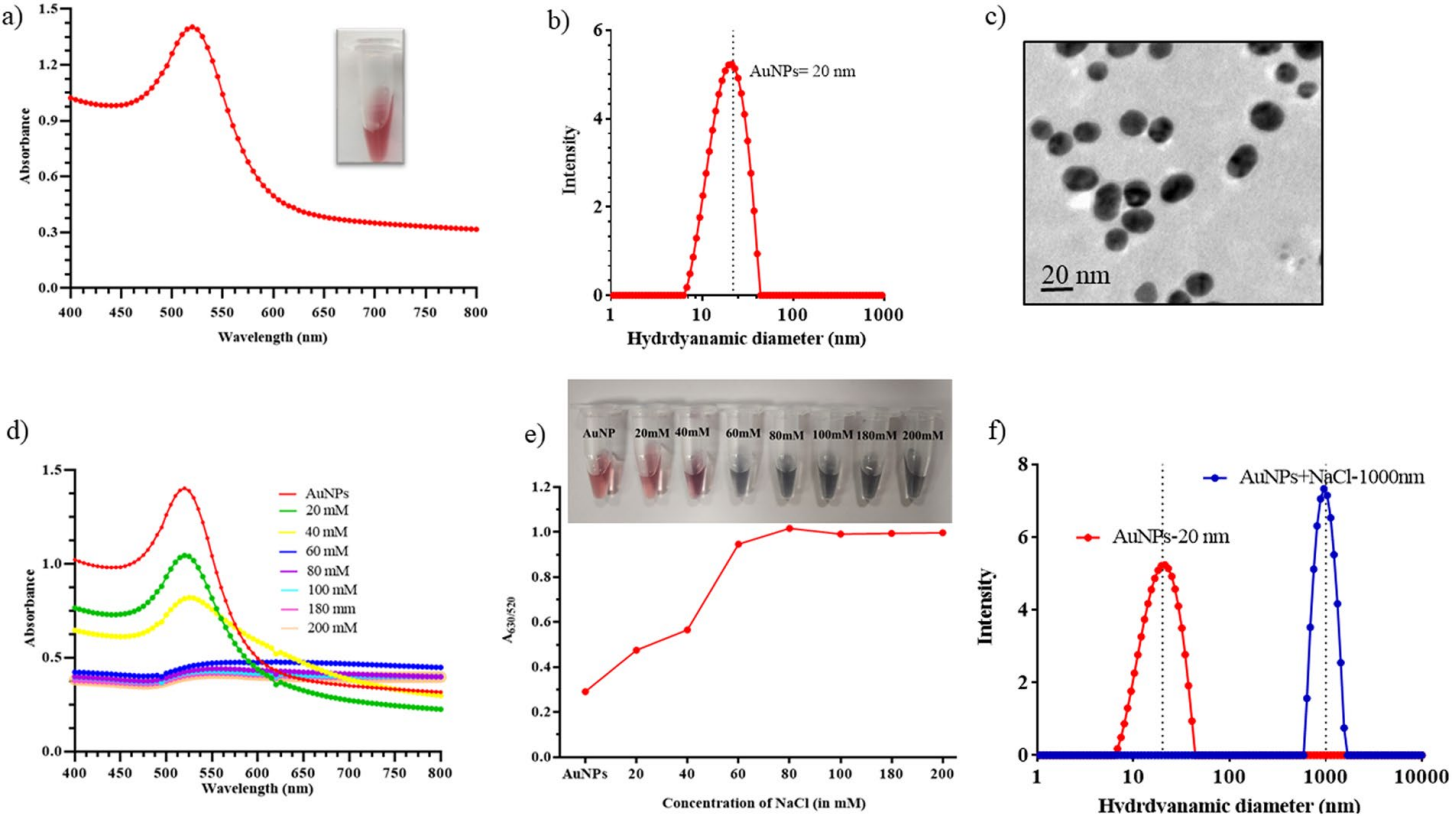
Characterization of AuNPs and optimization of NaCl concentration; (**a**) UV-Vis spectra of AuNPs with absorbance peak at 520 nm; (**b**) DLS showed the hydrodynamic diameter of AuNPs 20 ± 5 nm; (**c**) Monodispersed 20 nm AuNPs as depicted by TEM image; (**d**) UV-Vis absorption spectra of AuNPs with different concentrations of NaCl (20–200 mM); (**e**) Absorbance peak ratio of A_630/520_ nm of AuNPs with different concentration of NaCl and visual observation of AuNPs in different concentrations of NaCl; (**f**) Comparison of hydrodynamic diameter of dispersed AuNPs and aggregated AuNPs in the presence of NaCl.

18–20 nm AuNPs were synthesized according to the protocol described by Gandhi *et al*.^18^ and characterized using UV-Vis spectroscopy, TEM, and DLS. The UV-Vis wavelength scan was taken in the range of 400–800 nm. The major peak was observed at 520 nm that confirmed the size in the range of 15–20 nm (Fig. 1a). The hydrodynamic diameter of AuNPs was approximately 20 ± 5 nm with zeta potential of −38 mV (Fig. 1b). The morphological characteristics and dispersion of AuNPs analyzed with TEM, indicated a size of 18 ± 5 nm with fairly monodispersed particles (Fig. 1c). Aggregation of AuNPs was dependent on the concentration of NaCl. Stability of AuNPs was investigated using different concentrations of NaCl from 20 mM to 200 mM (Fig. 1d). At low concentration of NaCl (20 mM), aggregation was not induced, therefore no change in the colour of AuNPs was observed. Contrarily, increased concentration of NaCl (200 mM) affected the stability of AuNPs and caused aggregation. Absorption peak ratio of A_630/520_ increased with increase in the salt concentration as shown in Fig. 1e. Photographic image (inset Fig. 1e) shows the aggregation pattern at different concentrations of NaCl that started with 40 mM and attained saturation after 80 mM. Hydrodynamic diameter of the AuNPs increased from 20 nm to 1000 nm after aggregation in the presence of NaCl (Fig. 1f).

### Optimization of aptamer concentration

**Figure 2:**
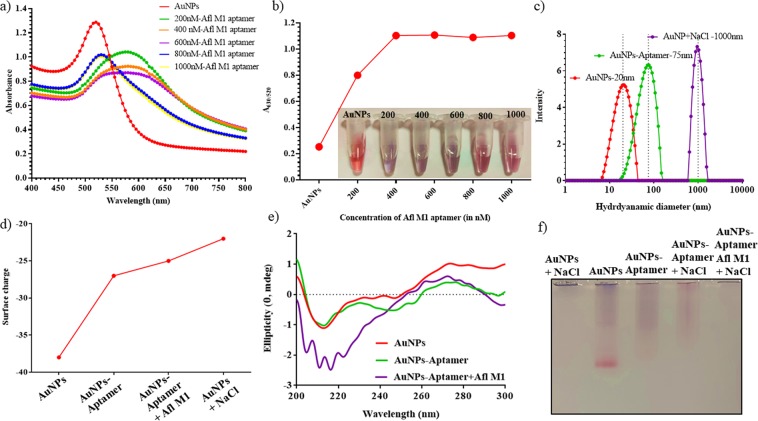
Optimization of different concentrations of Aflatoxin M1 aptamer; (**a**) UV-Vis absorption spectra of different concentrations of Afl M1 aptamer-modified AuNPs (200 nM–1000 nM) with 400 mM NaCl; (**b**) Absorbance peak ratio of A_630/520_ nm and visual observation of different concentrations of aptamer-modified AuNPs + 400 mM NaCl; (**c**) Hydrodynamic diameter of AuNPs, AuNPs/aptamer, and AuNPs-aptamer/Afl M1; (**d**) Zeta potential of AuNPs, Aptamer modified AuNPs, AuNPs-aptamer/Afl M1; (**e**) CD spectra of AuNPs, AuNPs/aptamer, and AuNPs-aptamer/Afl M1; (**f**) 0.8% agarose gel electrophoresis of AuNPs + NaCl, AuNPs, AuNPs/aptamer, AuNPs/aptamer + NaCl and AuNPs/aptamer/Afl M1 + NaCl.

Concentration of NaCl was optimized with AuNPs in the presence of Afl M1 aptamer. Optimum concentration of NaCl was determined by the shift in the absorbance peak ratio of A_630/520_ at different concentrations of Afl M1 aptamer. At different concentrations of aptamer (1000 nm- 200 nM) modified AuNPs and fixed 400 mM NaCl concentration, there was significance difference in the wavelength shift upto 600 nm of M1 aptamer (Fig. 2a). Absorbance peak ratio A_630/520_ increased with increase in aptamer concentration and reached saturation after 400 nM (Fig. 2b). It was observed that, size of AuNPs+NaCl decreased from 1000 nm to 75 nm in the presence of apt M1/AuNPs complex in comparison with AuNPs (20 nm) that confirmed the aggregation of AuNPs in presence of NaCl and further dispersion when AuNPs were protected with aptamer M1 (Fig. 2c). 800 nM AuNPs/Afl M1 aptamer concentration was chosen to optimize the concentration of salt. Zeta potential of AuNPs changed from −38 mV to −27 mV on addition of Afl M1 aptamer that indicated the reduction in electrostatic repulsion between the AuNPs (Fig. 2d). While analyzing CD spectra, it was assumed that the spectrum of an AuNP can be represented by a linear combination of the spectra of its secondary structural elements. AuNPs have zero ellipticity since they did not show any chiral properties (red line). However, ssDNA aptamer modified AuNPs samples presented a decrease in ellipticity, around 220 nm, due to their chiral properties (green line). The spectral difference between the AuNPs and aptamer-conjugated AuNPs showed the changes in the helix structures of the DNA molecules, indicating the successful bio modification of the nanoparticle surfaces. On addition of aflatoxin M1 to aptamer modified AuNPs, there is a shift in ellipticity at 210 nm of about 5–20 nm due to variation in the structure (voilet line) (Fig. 2e). AuNPs showed the highest mobility due to negative charge. Upon binding of aptamer M1 to AuNPs, mobility of the resulting complex moderately decreased. There was retardation in the mobility in the gel in case of aggregated AuNPs and aptamer modified AuNPs in the presence of Aflatoxin M1 (Fig. 2f).

### Quantitative detection of aflatoxin

**Figure 3:**
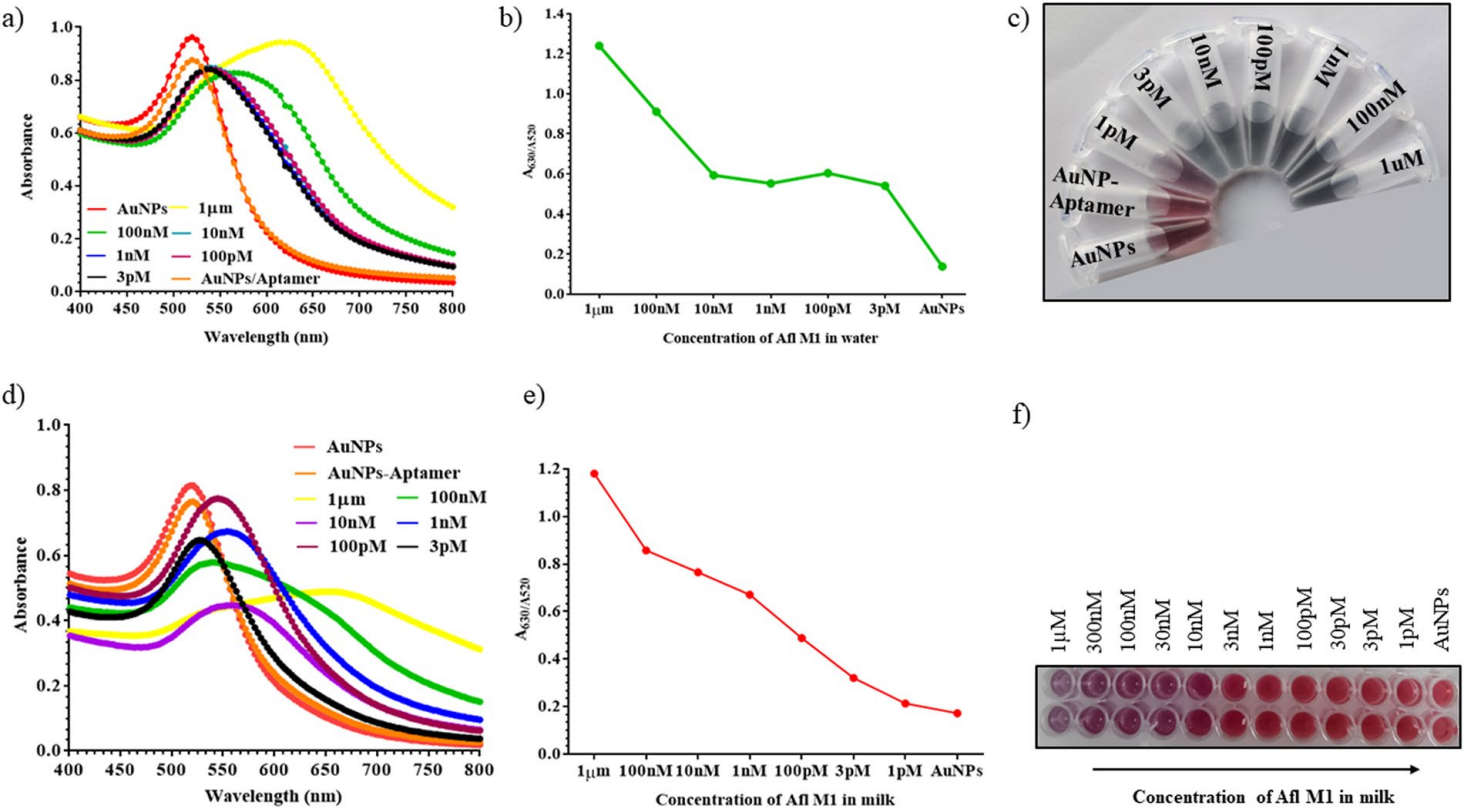
(**a**) UV-Vis spectra of aptamer modified AuNPs with different concentrations of Aflatoxin M1 in water (1 μM to 1 pM) and (**b**) Absorbance peak ratio at A_630/520_ nm; (**c**) Image of different concentrations of Aflatoxin M1 in water; (**d**) UV-Vis absorption spectra of aptamer modified AuNPs with different concentrations of Aflatoxin M1 spiked in milk (1 μM to 1 pM) and (**e**) Absorbance peak ratio of A_630/520_ nm; (**f**) Image of different concentrations of Aflatoxin M1 with aptamer modified AuNPs.

For quantitative analysis, Afl M1 concentrations were prepared ranging from 1 μM to 1 pM both in water and milk sample. UV-Vis spectroscopy, and A_630/520_ ratio was done in the presence of 400 mM NaCl and 800 nM Afl M1 aptamers (standardized as described earlier). Figure 3a,d. show the absorbance shift of AuNPs on addition of different concentrations of Aflatoxin M1 (1 μM to 1 pM) spiked in water and milk sample. Absorbance peak ratio (A_630/520_) increased with increasing concentrations of Afl M1, which correlated with enhanced displacement of Afl M1 aptamers conjugated with AuNPs in the presence of NaCl. High concentration of aptamers almost led to saturation with a flattened peak due to binding of complete aflatoxin M1 with its aptamer, leaving AuNPs to react with NaCl and which resulted in aggregation. At 1 µM concentration of Aflatoxin M1 that resulted in highest aggregation showed the absorbance peak ratio of 1.2 at A_630/520_ nm (Fig. 3b,e). It can be seen in Fig. 3c,f, limit of detection for Aflatoxin M1 in water was 3 pM and in spiked milk was 10 nM which was observed by naked eyes. The specificity of Aflatoxin M1 was cross checked with ochratoxin in water. There was no colour change observed when added at higher concentration of Ochratoxin. The Aflatoxin M1 aptamer was highly specific for Aflatoxin M1 but not for Ochratoxin as shown in Fig. S1(a,b).

### Fabrication and characterization of microfluidic paper device

**Figure 4:**
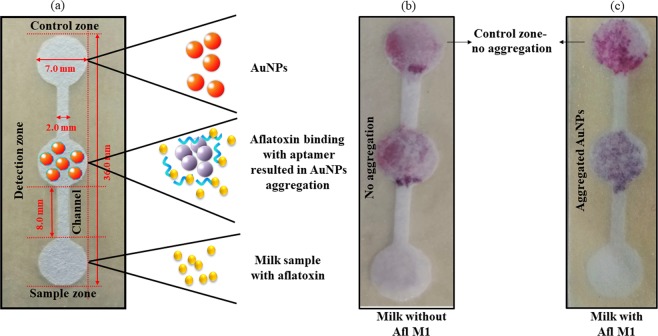
Microfluidic device (µPAD) for the detection of Aflatoxin M1 in milk; (**a**) Detailed dimensions of the paper based device; (**b**,**c)** Paper device in absence and presence of Aflatoxin M1.

Whatman filter paper was used for fabrication of the paper based microfluidic device. This μPAD consisted of three zones- detection, sample and control zone. Hydrophobic area was created using negative photoresist. The diameter of all three zones was 7.0 mm, width of the channel was 2.0 mm and height was 36.0 mm as shown in Fig. 4a. In detail, control zone contained of AuNPs, detection zone AuNPs/Afl M1 aptamer complex, and the sample zone contained Afl M1 spiked in milk (Fig. 4a). AuNPs and AuNPs/Afl M1 aptamer complex were loaded on the control and detection zones respectively and allowed to get adsorbed onto the designed paper pad by simple diffusion mechanism. Figure 4b,c showed that the milk spiked with Aflatoxin M1 caused aggregation of AuNPs conjugated with Afl M1 aptamer in the detection zone (colour changed from pink to blue) while in control zone there was no change in colour. The possible explanation to this phenomenon is the binding of Afl M1 with its aptamer that enhanced the displacement reaction. The ochratoxin aptamer was used for specificity and interference studies. Fig. S1.b-i,ii, showed the microfluidic paper device where milk samples were spiked with Aflatoxin M1 (sample zone) and test zone comprise of (b-i) Aflatoxin M1, and (b-ii) Ochratoxin. Due to non-specificity, there was no colour change in test zone (b-ii), while due to specificity of Aflatoxin M1 colour change was observed in sample zone (b-i).

Table 1 depicts the comparison between different detection techniques for aflatoxin M1. Most of the techniques are based on sensors that have lower limits of detection as compared to µPAD, however they are expensive and require regular calibration. On the other hand, the paper based microfluidic device developed in this study is easy to use, field applicable, rapid, and cost effective.

**Table 1:** Comparison of different techniques for the detection of Aflatoxin in milk.

S. No.	Name of the toxin	Type of technique	Biorecognition element	LOD	Reference
1.	Aflatoxin M1	Aptamer based biosensor	biotin–streptavidin	0.03 ng/l	^30^
2.	Aflatoxin M1	Electrochemical biosensor	(anti-AFM_1_)	0.05 μg/l	^31^
3.	Aflatoxin M1	Sweet sensor	IgGMS-M1	27 parts per trillion (ppt)	^32^
4.	Aflatoxin M1	ATR-FTIR spectroscopy	—	0.02 μg/l	^33^
5.	Aflatoxin M1	Fe_3_O_4_/polyaniline-based aptasensor	Magnetic nanoparticles	1.98 ng/l	^19^
6.	Aflatoxin M1	Background fluorescence quenching immunochromatographic assay (bFQICA)	GNPs-labeled antibody	0.0009 ng/ml	^34^
7.	Aflatoxin M1	µPAD	AuNPs conjugated aptamer	10.0 nM	This work

## Conclusion

In this work, a paper based microfluidic device was developed for the detection of aflatoxin M1 in water and milk. AuNPs were used as an indicator for the presence or absence of aflatoxin M1. The displacement reaction occurred in the presence of M1 aptamers that allowed aflatoxin to bind selectively with M1 aptamers. The presence of NaCl caused aggregation of AuNPs indicated by change in colour on the paper device from wine red to blue (both in the water and milk samples). The above said phenomenon was assessed thoroughly by UV-Vis spectroscopy (peak shift) and DLS (in terms of hydrodynamic diameter and zeta potential). Finally, the 21-mer M1 aptamer based μPAD showed a detection limit of 3 pM in water and 10 nM in milk samples. The developed paper based microfluidic device can be stored at room temperature for upto 3 months without any loss of activity and is a cost effective and rapid alternative to bulky, sophisticated instrumentation detection techniques.

## Supplementary information

Supplementary information.
