# Author Correction: Fabrication of microfluidic device for Aflatoxin M1 detection in milk samples with specific aptamers

**DOI:** 10.1038/s41598-020-64241-8

**Published:** 2020-06-03

**Authors:** Aruna Kasoju, Deepshikha Shahdeo, Azmat Ali Khan, Narlawar Sagar Shrikrishna, Subhasis Mahari, Amer M. Alanazi, Mashooq Ahmad Bhat, Jyotsnendu Giri, Sonu Gandhi

**Affiliations:** 1 DBT- National Institute of Animal Biotechnology, Hyderabad, 500032 India; 2 Department of Biotechnology, JNTUA College of Engineering, Andhra Pradesh, 516390 India; 3 Department of Pharmaceutical Chemistry, College of Pharmacy, Kind Saud University, Riyadh, 11451 Kingdom of Saudi Arabia; 4 Department of Biomedical Engineering, Indian Institute of Technology (IIT), Hyderabad, 502285 India

Correction to: *Scientific Reports* 10.1038/s41598-020-60926-2, published online 13 March 2020

The original version of this Article contained errors.

Affiliation 3, was incorrectly given as ‘Department of Pharmaceutical Chemistry, King Saud University, Riyadh, 11451, Saudi Arabia’. The correct affiliation is listed below:

Department of Pharmaceutical Chemistry, College of Pharmacy, King Saud University, Riyadh, 11451, Kingdom of Saudi Arabia.

Additionally, the title for Table 1 was incorrect.

‘Comparison of different techniques for the detection of Aflatoxin in mi.’

now reads:

‘Comparison of different techniques for the detection of Aflatoxin in milk.’

The Supplementary Information file contained a typographical error in the spelling of the author Azmat Ali Khan, which was incorrectly given as Azamat Ali Khan.

Additionally, in the Supplementary Information file,

“Figure S1. (a) Image of different concentration of Ochratoxin in water; (b) Microfluidic device (µPAD) for the detection of Aflatoxin M1 and Ochratoxin in milk; (i) presence of Aflatoxin M1aptamer led to aggregation of AuNPs and (ii) in presence of Ochratoxin aptamer, no aggregation occurred due to non-specificity with Aflatoxin M1.”

now reads:

“Figure S1. (a) Image of different concentration of Ochratoxin in water; (b) Microfluidic device (µPAD) for the detection of Aflatoxin M1 and Ochratoxin in milk; (i) presence of Aflatoxin M1aptamer led to aggregation of AuNPs and (ii) in presence of Ochratoxinaptamer, no aggregation occurred due to non-specificity with Aflatoxin M1.”

These errors have now been corrected in the HTML and PDF versions of the Article and in the Supplementary Information file that accompanies the article.

