# Supplementary material for: Fabrication of microfluidic device for Aflatoxin M1 detection in milk samples with specific aptamers
Source: Sci Rep. 2020 Mar 13;10:4627. doi: 10.1038/s41598-020-60926-2 (PMC7070014; doi:10.1038/s41598-020-60926-2)
Supplement: Supplementary file 1 — Supplementary information. [file 41598_2020_60926_MOESM1_ESM.docx]

**Supporting Information**

**Fabrication of microfluidic device for Aflatoxin M1 detection in milk samples with specific aptamers**

Aruna Kasoju^a,b^, Deepshikha Shahdeo^a^, Azmat Ali Khan^c^, Narlawar Sagar Shrikrishna^a^, Subhasis Mahari^a^, Amer M. Alanazi^c^, Mashooq Ahmad Bhat^c^, Jyotsnendu Giri^d^, Sonu Gandhi^a,^**^#^**

^a^DBT- National Institute of Animal Biotechnology, Hyderabad-500032, India

^b^Department of Biotechnology, JNTUA College of Engineering, Andhra Pradesh- 516390, India

^c^Department of Pharmaceutical Chemistry, College of Pharmacy, King Saud University, Riyadh-11451, KSA

^d^Department of Biomedical Engineering, Indian Institute of Technology (IIT), Hyderabad- 502285, India

**^#^Corresponding address:**

**Dr. Sonu Gandhi**; E-mail:[sonugandhi@gmail.com](mailto:sonugandhi@gmail.com); [gandhi@niab.org.in](mailto:gandhi@niab.org.in)


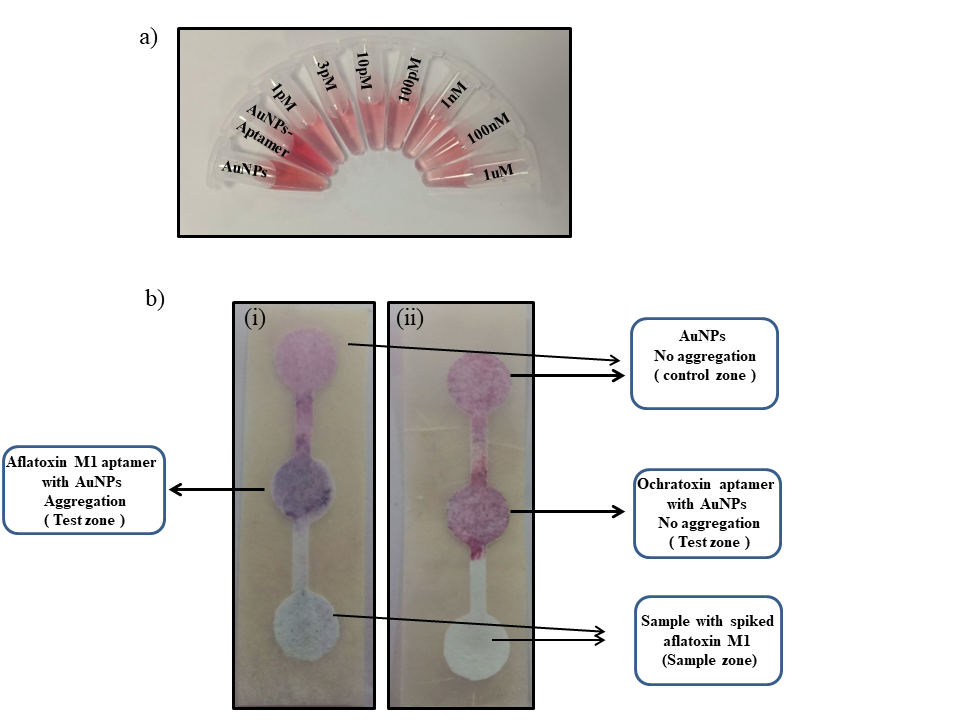
Figure S1. (a) Image of different concentration of Ochratoxin in water; (b)Microfluidic device (µPAD) for the detection of Aflatoxin M1 and Ochratoxin in milk; (i) presence of Aflatoxin M1aptamer led to aggregation of AuNPs and (ii) in presence of Ochratoxinaptamer, no aggregation occurred due to non-specificity with Aflatoxin M1.
